# Effects of Interface Pressure Distribution on Human Sleep Quality

**DOI:** 10.1371/journal.pone.0099969

**Published:** 2014-06-12

**Authors:** Zongyong Chen, Yuqian Li, Rong Liu, Dong Gao, Quanhui Chen, Zhian Hu, Jiajun Guo

**Affiliations:** 1 Sleep Research Center, DaZiRan Science and Technology Ltd., Guiyang, Guizhou, China; 2 Department of Physiology, Third Military Medical University, Chongqing, China; 3 Department of Sleep and Psychology, Institute of Surgery Research, Daping Hospital, Third Military Medical University, Chongqing, China; Peking University, China

## Abstract

High sleep quality promotes efficient performance in the following day. Sleep quality is influenced by environmental factors, such as temperature, light, sound and smell. Here, we investigated whether differences in the interface pressure distribution on healthy individuals during sleep influenced sleep quality. We defined four types of pressure models by differences in the area distribution and the subjective feelings that occurred when participants slept on the mattresses. One type of model was showed “over-concentrated” distribution of pressure; one was displayed “over-evenly” distributed interface pressure while the other two models were displayed intermediate distribution of pressure. A polysomnography analysis demonstrated an increase in duration and proportion of non-rapid-eye-movement sleep stages 3 and 4, as well as decreased number of micro-arousals, in subjects sleeping on models with pressure intermediately distributed compared to models with over-concentrated or over-even distribution of pressure. Similarly, higher scores of self-reported sleep quality were obtained in subjects sleeping on the two models with intermediate pressure distribution. Thus, pressure distribution, at least to some degree, influences sleep quality and self-reported feelings of sleep-related events, though the underlying mechanisms remain unknown. The regulation of pressure models imposed by external sleep environment may be a new direction for improving sleep quality. Only an appropriate interface pressure distribution is beneficial for improving sleep quality, over-concentrated or -even distribution of pressure do not help for good sleep.

## Introduction

Sleep occupies one third of our lifetime and plays an important role in many physiological functions, such as learning and memory, metabolism, immunity, cardiovascular regulation [1], [2], [3], [4]. Recent studies also indicated a role of sleep in cellular homeostasis and clearance of metabolic wastes [5], [6]. Compelling evidence suggests that sleep deprivation gives rise to deficiencies in these functions [7], [8], [9]. Sleep quality and related functions are regulated by internal neural networks [10] and external environmental factors, such as temperature [11], light [12], sound and smell [13], [14], [15]. Previous studies revealed that patients with obstructive sleep apnea experience a better sleep, with higher total sleep time and sleep efficiency, under a room temperature of 16°C compared to 24°C [11]. Additionally, an inappropriate external environment can lead to sleep disorders, such as, insomnia. Abnormal sleep quality gives rise to deteriorated emotions and an inefficiency in performance during the following day [16]. Pressure sensation is one of the most important interactions between subjects and the external environment. To date, the effects of interface pressure distribution on sleep quality have not attracted much attention.

It is reasonable to speculate that different mattresses will impose different models of pressure on subjects during sleep. Previous studies have demonstrated that the maximum or average pressures were higher when patients with spinal cord injuries slept on a Dynamic Flotation System mattress compared to a Pegasus Airwave mattress [17]. Similarly, a low airloss surface significantly reduced the interface pressure compared with standard hospital innerspring mattress or a foam mattress [18]. In addition, different parts of the body are supported by the mattress, with the greatest pressure on the pelvic area and the shoulder [19], [20]. Body position also plays a role in the distribution of contact pressure [21], [22]. We explored whether the pressure distribution can be defined by mattresses.

To characterize the role of the interface pressure distribution in sleep, we detected the maximum and minimum pressure, the total stressed area and the area of distribution at different pressure levels. Additionally, we used polysomnography (PSG) recording to investigate whether sleep quality was influenced by the differences in the pressure distribution models. Questionnaires were used to further explore the role of pressure distribution in the subjective feelings of sleep.

## Materials and Methods

### Participants

Sixteen healthy male adults were recruited. Subjects ranged in age from 20 to 45 years, with a Midn height, weight, and body mass index of 166.35 cm, 61.70 kg, and 22.30 kg/m^2^, respectively. Self-reports indicated that none of the subjects had sleep disorders such as sleep apnea; all vital signs, such as respiratory rate, heart rate, blood pressure, and body temperature, were within the normal ranges.

### Materials

An AliceLE PSG was purchased from the Philips Company and used to record the sleep quality. The PSG includes 2 electroencephalography (EEG) channels, 2 electrooculography channels (EOG), and 2 electromyography channels (EMG). The EEG data were analyzed in every 30-second epoch according to *The AASM Manual for the Scoring of Sleep and Associated Events Rules, Terminology and Technical Specifications*. The position sensor was fixed on the midline of the chest and used for recording the number of turn-overs during sleep. An ABW BPMS Research system was used for the measurement of the pressure that supports different parts of the body in different postures. A total of 684 independent pressure sensors, with a measuring range of 0 to 75 mm Hg, were arranged in approximately 2 m^2^. To test whether the sleep quality was influenced by the conditions of the sleep mediums that created different pressure distribution models during sleep, we used four types of mattresses purchased from the DaZiRan Science and Technology Ltd. in Guizhou. Mattress I and IV were made of plank and independent springs, respectively. Mattress II was composed of a supporting layer and a pillow top and was made of palm fiber. Mattress III was a 3D structure mattress and was made of foam rubber and plant fiber; apart from the supporting layer and pillow top, this mattress had another layer between these two layers that finely fit the shape of the human body. Finally, three infrared cameras were used to simultaneously record the behaviors of the subjects during sleep.

### Procedures

These experiments were approved by the Ethics Committee of the *DaZiRan* Science and Technology Ltd., and oral informed consent was given to every participant, after which a written form was obtained. Subjects were asked to abstain from smoking and to avoid strenuous exercise and stimulatory agents, such as coffee and wine, during the daytime prior to the experiment. The room temperature was maintained at 25°C during sleep.

Subjects were given detailed instructions on the procedures of the experiments. They arrived at the laboratory at 20:00 and spent one hour to familiarize the recording environment. A self-report of previous sleep quality, including the times at which the participants goes to bed and gets up, the temperature, the humidity, the light, the noise, the smell of the sleep environment, the mattress types, the dreams and the subjective feelings of sleep were obtained from every participant prior to the first trial. The distribution area of pressure at different parts of the body while lying in a flat and on one side on a specific mattress was measured. In the supine position, participants lay on the mattress with face up; hands put on both sides of the body; legs keep straight. In lateral position, participants lay on one side on the mattress with the legs slightly bent; hands placed naturally. Electrodes were placed on the head in accordance with the international 10–20 system. EEGs were recorded from frontal derivations (F3 and F4); electrodes for EOG recording were placed 1 cm lateral-superior (right) and lateral-inferior (left) to the outer canthus of each eye, with 2 reference electrodes on M1 and M2. EMGs were recorded from 2 cm to the midline of the chin. The PSG recording was begun at 9:00 p.m.−10:00 p.m. with the indication of a ringing bell given by the participants, which was followed by biological calibration. The recording was terminated at 6:00 a.m.−7:00 a.m. when the subjects woke up in the next morning. Finally, the subjects were asked to complete a post-experiment questionnaire elated to the self-evaluation of sleep quality during the recording. Order of mattresses was randomly assigned, double-blind design was used during all the studies and 2 days were assigned as blanking period between two trails. All subjects included in the statistical analysis finished the test of the four types of mattresses and corresponding enquiries in two weeks.

### Statistical Analysis

Values were presented as mean ± S.E.M. All analyses were conducted using SigmaPlot 11.0. One way analysis of variance (ANOVA), Kruskal-Wallis one way ANOVA on Ranks, least significant difference (LSD) test, Student-Newman-Keuls (SNK) test, paired t test, Chi-square test and Wilcoxon signed rank test were used for the statistical analyses. A *P* value less than 0.05 was considered statistically significant for all tests.

## Results

### Four Models of Interface Pressure Distribution were Identified by the Different Mattresses

**Figure 1:**
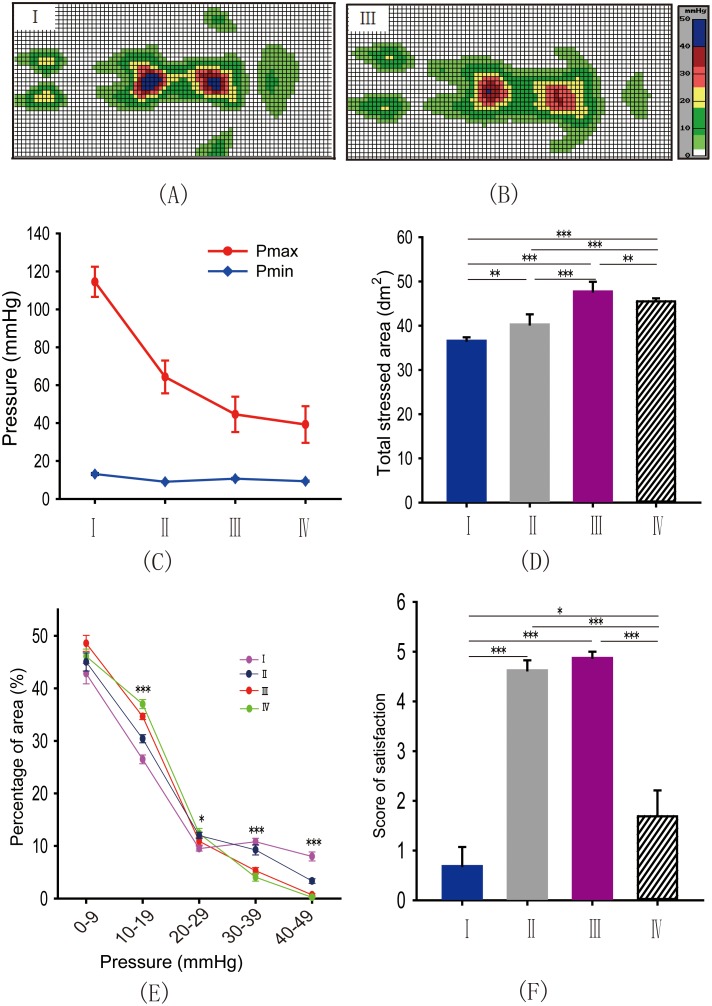
Four models of interface pressure distribution were identified by the different mattresses. (A, B) Examples of the distribution area of pressure at different levels on two models I and III. (C, D) The mean Pmax, Pmin and total stressed area were significantly different between the four mattresses. (One way ANOVA and LSD test, Maximum pressure: *P*_I*IV_<0.05, *P*_I*II_<0.001, *P*_I*III_<0.001, *P*_II*IV_<0.05; Minimum pressure: *P*_I*IV_<0.001, *P*_I*II_<0.001, *P*_I*III_<0.001, *P*_II*III_<0.001; Total stressed area: *P*_I*IV_<0.001, *P*_I*II_<0.01, *P*_I*III_<0.001, *P*_II*IV_<0.001, *P*_III*IV_<0.01, *P*_II*III_<0.001). (E) Significant differences in the percentage of area in blocks of 10–19 mm Hg, 20–29 mm Hg, 30–39 mm Hg and 40–49 mm Hg existed between the four mattresses. (One way ANOVA and LSD test, 10–19 mm Hg: *P*I*IV<0.001, *P*_I*II_<0.001, *P*_I*III_<0.001, *P*_II*IV_<0.001, *P*_III*IV_<0.05, *P*_II*III_<0.001; 20–29 mm Hg: *P*_I*IV_<0.01, *P*_I*II_<0.05; 30–39 mm Hg: *P*_I*IV_<0.001, *P*_I*II_<0.001, *P*_III*IV_<0.001, *P*_II*III_<0.001; 40–49 mm Hg: *P*_I*IV_<0.05, *P*_I*II_<0.05, *P*_I*III_<0.05, *P*_II*IV_<0.05, *P*_II*III_<0.05); (F) Subjects showed higher scores of satisfaction for mattresses II and III compared to I and IV, but with no significant differences between mattresses II and III (Kruskal-Wallis one way ANOVA on Ranks and SNK test, *P*_I*II_<0.001, *P*_I*III_<0.001, *P*_I*IV_<0.05, *P*_III*IV_<0.001, *P*_II*IV_<0.001). *P<0.05; **P<0.01; ***P<0.001.

To achieve different interface pressure distribution models, we used four types of mattresses, I II, III and IV to detect the maximum pressure (Pmax), the minimum pressure (Pmin) and the total stressed area (Ats) when subjects laid on these mediums (Fig. 1A, 1B). One way ANOVA and LSD test showed that the maximum Pmax and Pmin were experienced when subjects slept on mattress I (Figure 1C, Maximum pressure: *P*_I*IV_<0.05, *P*_I*II_<0.001, *P*_I*III_<0.001, *P*_II*IV_<0.05; Minimum pressure: *P*_I*IV_<0.001, *P*_I*II_<0.001, *P*_I*III_<0.001, *P*_II*III_<0.001), but the Ats was minimal in this condition (Fig. 1D, *P*_I*IV_<0.001, *P*_I*II_<0.01, *P*_I*III_<0.001, *P*_II*IV_<0.001, *P*_III*IV_<0.01, *P*_II*III_<0.001),, suggesting an “over concentrated” pressure distribution model. Oppositely, mattress IV produced the minimum Pmax and Pmin with large Ats, indicating an “over even” distributed pressure. The other two models were demonstrated “intermediate distribution” of pressure. Thus, the mattress type did significantly affect the Pmax, the Pmin or the Ats. Next, we further analyzed whether the area of distribution at different pressure levels was different when subjects sleeping on the four types of mattresses. An ANOVA showed significant differences in the area of distribution at 10–19 mm Hg, 20–29 mm Hg, 30–39 mm Hg and 40–49 mm Hg between the four types of mattresses (Fig. 1E, 10–19 mm Hg: *P*_I*IV_<0.001, *P*_I*II_<0.001, *P*_I*III_<0.001, *P*_II*IV_<0.001, *P*_III*IV_<0.05, *P*_II*III_<0.001; 20–29 mm Hg: *P*_I*IV_<0.01, *P*_I*II_<0.05; 30–39 mm Hg: *P*_I*IV_<0.001, *P*_I*II_<0.001, *P*_III*IV_<0.001, *P*_II*III_<0.001; 40–49 mm Hg: *P*_I*IV_<0.05, *P*_I*II_<0.05, *P*_I*III_<0.05, *P*_II*IV_<0.05, *P*_II*III_<0.05;). At higher pressure ranges, such as 30–39 mm Hg or 40–49 mm Hg, highest proportion of area was observed in mattress I but lowest in mattress IV. In contrast, highest percentage of area at low pressure ranges, such as 10–19 mm Hg and 20–29 mm Hg, was found in mattress IV while lowest in mattress I. Moreover, we investigated whether the differences between pressure distribution models would bias the subjective feelings about these mattresses. Surprisingly, Kruskal-Wallis one way ANOVA on Ranks and SNK test demonstrated that the participants reported higher scores of satisfaction for mattress II and III in the post-experiment reports (Fig. 1F, *P*_I*IV_<0.05, *P*_I*II_<0.001, *P*_I*III_<0.001, *P*_II*IV_<0.001, *P*_III*IV_<0.001). The data provided above indicated that different mattresses provide distinct areas of distribution and pressure support to the subjects, which may contribute to different sensations and feelings during sleep. According to the pressure distribution and subjective satisfaction, we artificially defined four types of pressure distribution models, that is M_oc_, M_id_, M_id_′, M_oe_. M_oc_ was produced by mattress I, and was showed “over-concentrated” pressure distribution; M_id_ and M_id_′ were represented by mattress II and III, and was displayed “intermediate distribution” of pressure; M_oe_ was generated by mattress IV, and was demonstrated “over-even” distributed pressure.

### Objective Sleep Quality under Different Pressure Models

**Figure 2:**
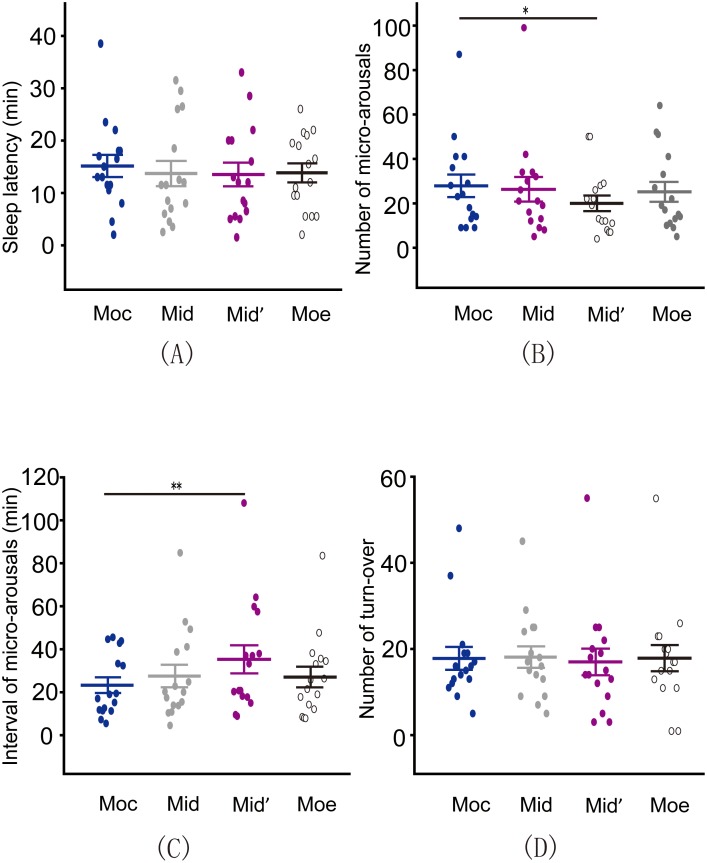
Micro-arousals were significantly decreased in M_id_′ compared to M_oc_. (A) Sleep latency was not influenced by differences in the pressure distribution models (One way ANOVA, *P*>0.05). (B, C) The number of micro-arousals was significantly decreased (Paired t test, *P*<0.05) and the mean interval between micro-arousals was increased (Wilcoxon signed rank test, *P*<0.01) on the M_id_′ compared to the M_oc_. (D) No significant differences in the number of turn-overs were detected between these four models (One way ANOVA, *P*>0.05). *P<0.05; **P<0.01.

**Figure 3:**
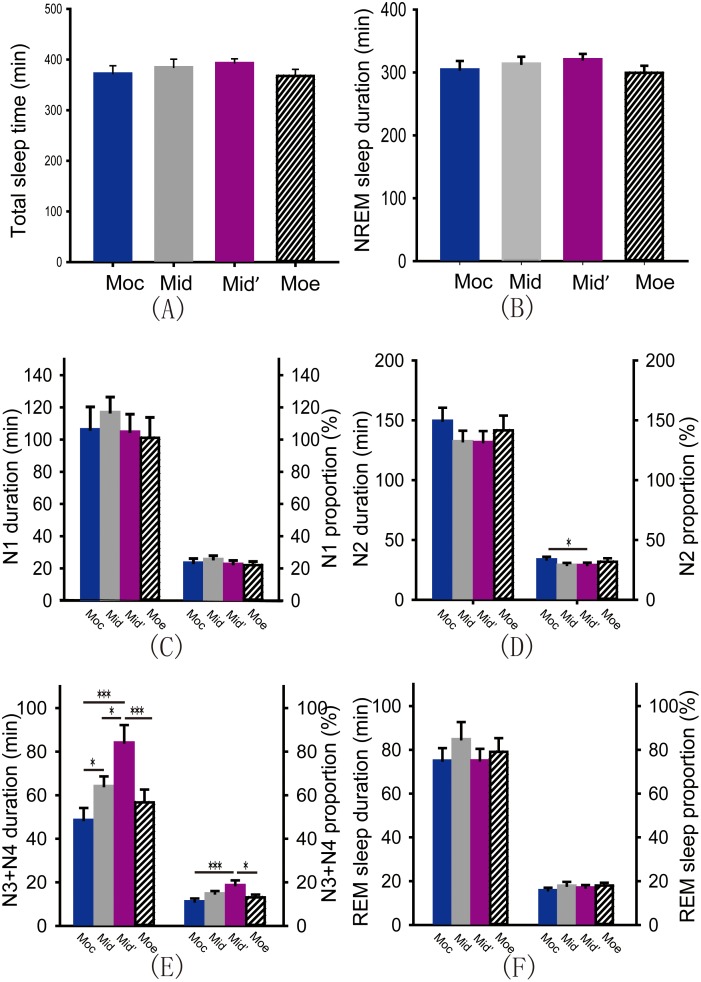
Duration and proportions of different sleep stages under four kinds of pressure distribution models. (A) Total sleep time (all *P*>0.05). (B) NREM sleep duration (all *P*>0.05). (C) Duration and proportion of N1 (all *P*>0.05). (D) Duration and proportion of N2 (Paired t test, *P*_Moc*Mid′_<0.05). (E) Duration and proportion of N3+N4. (One way ANOVA and LSD test, duration: *P*_Moc*Mid_<0.05, *P*_Moc*Mid′_<0.001, *P*_Moe*Mid′_<0.001, *P*_Mid*Mid′_<0.05; proportion: *P*_Moc*Mid′_<0.001, *P*_Moe*Mid′_<0.05). (F) Duration and proportion of REM sleep (all *P*>0.05). **P<0.01; *P<0.05; ***P<0.001.

**Figure 4:**
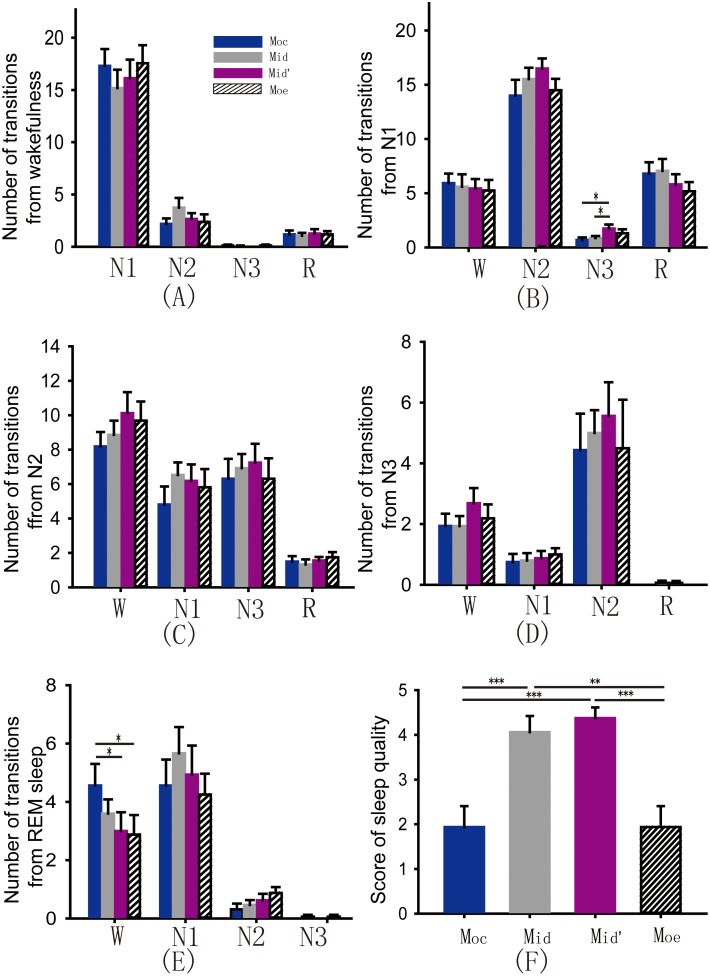
Number of transitions of sleep-wake stages and self-reported sleep quality. (A) Number of transitions from wakefulness to N1, N2, N3 and REM sleep (all *P*>0.05). (B) Number of transitions from N1 to wakefulness, N2, N3 and REM sleep (N1 to N3: *P*_Moc*Mid′_<0.05, *P*_Mid*Mid′_<0.05). (C) Number of transitions from N2 to wakefulness, N1, N3 and REM sleep (all *P*>0.05). (D) Number of transitions from N3 to wakefulness, N1, N2 and REM sleep (all *P*>0.05). (E) Number of transitions from REM sleep to wakefulness, N1, N2, and N3 (REM sleep to wakefulness: *P*_Moc*Mid′_<0.05, *P*_Moc*Moe_<0.05). (F) Self-reported score of sleep quality (*P*_Moc*Mid_<0.001, *P*_Moc*Mid′_<0.001, *P*_Moe*Mid_<0.01, *P*_Moe*Mid′_<0.001).

Sleep fragmentation leads to inefficient sleep quality [23]. To date, the role of pressure distribution on sleep fragmentation remains unknown. We analyzed the sleep latency, the number and intervals of micro-arousals, and the number of turn-overs in these four models. According to the results from the PSG recording, we found differences in the sleep latency under different models and in the number of turn-overs during the night under different models by one way ANOVA; however, these differences were not significant (Fig. 2A and 2D, *P*>0.05). Paired t test showed that number of micro-arousals was significantly decreased on the M_id_′ compared to the M_oc_ (Fig. 2B, *P*<0.05). The meann interval between the micro-arousals was consistently longer when subjects slept on the M_id_′ compared to the M_oc_ (Fig. 2C, Wilcoxon signed rank test, *P*<0.01). Next, we analyzed the subjects’ sleep stage distribution and transitions under these four types of pressure distribution models by PSG recordings. Surprisingly, one way ANOVA revealed that the total sleep time, duration of non-rapid eye movement (NREM) sleep, NREM sleep stage 1 (N1) and N2 and rapid eye movement (REM) sleep were not influenced by difference in the pressure distribution models (Figure 3A, 3B, 3C, 3D, 3F, all *P*>0.05). However, one way ANOVA and LSD test showed that the amount of time spent in N3 and N4 was highest when subjects slept on the M_id_′ and decreased successively when participants slept on the M_id_, the M_oe_ and the M_oc_ (Fig. 3E, *P*_Moc*Mid_<0.05, *P*_Moc*Mid′_<0.001, *P*_Moe*Mid′_<0.001, *P*_Mid*Mid′_<0.05). Further analysis showed that proportion of N3 and N4 was increased, but proportion of N2 was decreased, in M_id_′ compared to M_oc_ (Figure 3D, *P*_Moc*Mid′_<0.05; 3E, *P*_Moe*Mid′_<0.05; *P*_Moc*Mid′_<0.001). Finally, transitions between stages of sleep and wakefulness were calculated according to the previous reported methods [24]. Comparing to M_oc_, M_id_′ significantly decreased the number of transitions from REM sleep to wakefulness, and increased number of transitions from N1 to N3 (Figure 4B, *P*_Moc*Mid′_<0.05, *P*_Mid*Mid′_<0.05; 4E, *P*_Moc*Mid′_<0.05, *P*_Moc*Moe_<0.05). Other sleep stage transitions were not influenced by different pressure models (Figure 4A, 4C, 4D, all *P*>0.05). The above data demonstrated that the number of micro-arousals and transitions from REM sleep to wakefulness were decreased; duration and proportion of N3 and N4, transitions from N1 to N3 were increased under the M_id_′, suggesting that different models of pressure distribution have a significant impact on sleep quality, and M_id_′ is most appropriate for improving sleep quality.

### Self-reported Sleep Quality Under Four Types of Pressure Distribution Models

To further evaluate the subjective feelings of the four types of pressure models, we asked participants to finish a post-experiment enquiry regarding the sleep quality; statistical analyses were accomplished using non-parametric statistics. Chi-square test showed that subjects reported significant differences between the models, indicating that M_id_′ and M_id_ are softer than M_oc_ and M_oe_ (Table 1, Hard: *P*_Moc*Mid_<0.001, *P*_Moc*Mid′_<0.001; Soft: *P*_Moe*Mid_<0.001, *P*_Moe*Mid′_<0.001); this indicates a more comfortable situation at some degree. However, the subjective feelings between the M_id_ and the M_id_′ were not significant. Next, we investigated self-reported feelings regarding the difficulty to fall asleep on these different models. The results showed that only the M_id_ and the M_oc_, but not other models, were associated with differences in the self-reported difficulty of falling asleep (Table 1, Chi-square test, *P*_Moc*Mid_<0.01). Subjects fell asleep more easily when they slept on the M_id_ compared to the M_oc_. Finally, we asked participants to quantitate their feelings of sleep quality. As expected, one way ANOVA and LSD test suggested that participants’ feelings related to the sleep quality on the four models were significantly different (Fig. 4F, *P*_Moc*Mid_<0.001, *P*_Moc*Mid′_<0.001, *P*_Moe*Mid_<0.01, *P*_Moe*Mid′_<0.001), with higher scores for the M_id_′ and the M_id_ compared to the M_oe_ and the M_oc_.

**Table 1:** Subjective feelings when sleeping on different pressure distribution models.

	Hard		Soft		Comfortable		Difficult to fall asleep	
	Yes	No	Yes	No	Yes	No	Yes	No
Moc	15	1					8	8
Moe			12	4			5	11
Mid	1	15	0	16	15	1	1	15
Mid′	0	16	2	14	14	2	3	13
	P_Moc*Mid_<0.001		P_Moe*Mid_<0.001				P_Moc*Mid_<0.01	
	P_Moc*Mid′_<0.001		P_Moe*Mid′_<0.001					

## Discussion

In this study, we found that sleep efficiency is higher when subjects experience an appropriate distributed interface pressure during sleep, over-concentrated or over-even distribution of pressure is not beneficial to improve sleep quality.

Previous studies have demonstrated that sleep-quality is different when subjects slept on two types of mattress, though these results tended to be individual-specific [25]. Participants reported improved subjective feelings related to sleep quality, back discomfort and spine stiffness when they slept on a new type of bedding system compared to a typical mattress [26], [27]. Similarly, patients with back pain and sleep disorders had significant improvements when they slept on a special mattress made of foam and latex [28]. In 2006, Hyunja and colleagues investigated the influence of mattress types on sleep quality and skin temperature. They found that when subjects slept on a self-reported “comfortable” mattress, the sleep efficiency, the duration of N3 and N4, and the skin’s temperature were higher than when subjects slept on an “uncomfortable” mattress [29]. Moreover, different bedding systems produced different sleep-related respiratory disturbances and slow-wave sleep [30]. These experiments did not focus on the role of pressure distribution in sleep quality. Our results further identified that different models of pressure significantly biased the efficiency of sleep.

Sensory input plays an important role in neuronal activities during sleep, though the sensory threshold is largely increased during this process. As mentioned above, the temperature [11], light [12], sound and smell [13], [14], [15] around the environment will bias the architecture of the individual’s sleep pattern and related functions. Recently, Ngo and colleagues applied an in-phase auditory stimulation to investigate the effects of sound on sleep and memory. Surprisingly, they found that the slow oscillations during sleep were significantly enhanced, and the subjects displayed improved performance in tasks related to declarative memory [13]. The pressure distribution on the human body during sleep is another aspect of sensory input that has received little attention to in previous years. Pressure sensations and the related signals are sent to the central nervous system and influence neuronal activity, and the patterns of the brain activity, during sleep. According to the results from the interface pressure distribution models and the self-reported satisfaction, the participants preferred models with appropriate distributed pressure. Because “over-concentrated” or “over-even” pressure distribution may influence the sensory input and disturb sleep-related brain networks or may change the physiological posture for better sleep. Whether the pathways and the neural networks related to the integration of sensory input are different in wakefulness and sleep should be clarified.

To make things more complicated, the mechanisms underlying the effects of pressure distribution on sleep quality remain largely unknown. We found that duration and proportion of N3 and N4 were increased, proportion of N2 was decreased, when subjects slept on the M_id_′ compared to M_oc_. The number of micro-arousals was also significantly higher with much shorter mean intervals between micro-arousals on the M_oc_ compared to the M_id_′. Transitions from REM sleep to wakefulness were decreased and transitions from N1 to N3 were increased in M_id_′ than M_oc_. Intriguingly, pressure models did not influence sleep latency, total sleep time, duration of N1 and N2 and REM sleep or number of turnovers. Thus, the sleep fragmentation decreased on the M_id_′ and the M_id_. An appropriate distributed pressure may be beneficial for damping neuronal perturbations, which may further facilitate the synchronization of neuronal groups, thereby increasing the duration of slow wave sleep.

We found a new aspect for improving sleep quality and related functions, especially memory consolidation. Patients with primary insomnia exhibit impairments in working memory and abnormal activity of task-associated brain regions, such as the dorsolateral prefrontal cortex [31]. Our previous studies have also demonstrated that short-term sleep deprivation influences the excitability of neurons in the prefrontal cortex [32]. Moreover, adenosine, the intrinsic sleep-promoting factor, plays a role in the regulation of neuronal excitability in the entorhinal cortex [33], a brain region involved in learning and memory [34], [35]. Because slow wave sleep is beneficial for the consolidation of declarative memories [36], [37], our results imply that the regulation of pressure distribution may be effective in improving the performance of subjects in tasks related to declarative memory, though behavioral studies have not yet been conducted. Thus, exploring the optimal pressure distribution models with other environmental factors holds promise for greatly increasing the efficiency of sleep.
